# “Tension Suspension” Remnant‐Preserving ACL Reconstruction Promotes the Early Maturity of the Graft Ligament

**DOI:** 10.1111/os.70443

**Published:** 2026-10-14

**Authors:** Jiamin Liang, Gengen Huang, Wenhan Yang, Junming Wei, Yingzhen Yang, Zirong Huang, Weimin Zhu

**Affiliations:** ^1^ Department of Medicine Shenzhen University Shenzhen Guangdong Province China; ^2^ Department of Sports Medicine The Second People's Hospital of Shenzhen City, The First Affiliated Hospital of Shenzhen University Shenzhen Guangdong Province China

**Keywords:** ACL reconstruction, femoral tunnel enlargement, graft healing, ligamentization, remnant preservation

## Abstract

**Purpose:**

The clinical application of anterior cruciate ligament (ACL) remnant preservation remains controversial, and conventional remnant‐preserving techniques cannot fully maximize the biological potential of ACL remnants. To compare graft ligamentization, femoral tunnel enlargement, and clinical functional scores between “tension suspension” remnant‐preserving anterior cruciate ligament reconstruction (ACLR) and single‐bundle anatomical ACLR.

**Methods:**

Forty‐eight patients enrolled from December 2021 to June 2023 were divided into non‐preserving remnant (NPR, *n* = 18) and preserving remnant (PR, *n* = 30) groups. At 3 and 6 months postoperatively, magnetic resonance imaging (MRI) of the knee was performed to measure graft signal noise quotient (SNQ) of the region of interest (ROI) and femoral tunnel diameter changes. Lysholm, IKDC, and Tegner scores were assessed at 6 months. Demographic and preoperative data were analyzed; intergroup comparisons of graft SNQ, femoral tunnel diameter changes, and clinical scores were conducted. Spearman correlation analysis evaluated the relationship between graft SNQ and knee function scores.

**Results:**

No intergroup differences were noted in age, sex, BMI, or graft diameter. At 3 months, significant differences were observed in graft SNQ (most prominent at the distal end) and femoral tunnel diameter changes (*p* < 0.05). At 6 months, no significant differences were found in SNQ (except near‐significant proximal SNQ, *p* = 0.058) or femoral tunnel diameter changes; clinical scores showed no intergroup differences. Tegner scores were moderately to significantly positively correlated with 6‐month SNQ (< 0.05).

**Conclusion:**

“Tension suspension” remnant‐preserving ACLR has significant advantages in early graft ligamentization and inhibiting femoral tunnel enlargement. Subjective knee function may be related to graft signal intensity.

## Graphical abstract

The manuscript compared the “tension suspension” remnant‐preserving ACL reconstruction with traditional single‐bundle anatomical reconstruction in terms of graft ligamentization, femoral tunnel enlargement, and clinical functional scores. The results demonstrated that the remnant‐preserving surgical approach showed significant advantages in early graft ligamentization and femoral tunnel stability. This research provides important experimental data and clinical evidence for optimizing ACL reconstruction surgery.

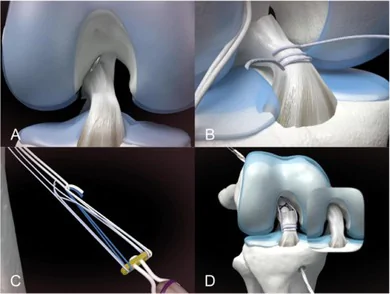

## 1 Introduction

Anterior cruciate ligament (ACL) injury is a common sports injury [1]. ACL rupture leads to anterior and rotational instability of the knee joint, and long‐term knee joint instability leads to secondary meniscus injury, ultimately progressing to knee osteoarthritis [2]. Currently, arthroscopic anterior cruciate ligament reconstruction (ACLR) is the gold standard for the treatment of ACL rupture [3]. The most commonly used graft in China is the hamstring tendon [3]. With the increase in the number of patients undergoing ACLR, researchers have paid widespread attention to postoperative return to sport, graft failure, and rerupture of ACLR [4, 5]. The rupture of grafts causes enormous psychological and physiological pressure, as well as a significant medical burden for patients.

At present, research suggests that risk factors for secondary rupture include nonclosure of epiphyseal plates [6, 7], poor ligament formation of grafts (joint cavity graft ligament plasticity, tendon bone healing), enlargement of bone tunnels [8], failure of graft ligament fixation, and incorrect positioning of bone tunnels [9]. Therefore, exploring methods to promote the process of graft ligamentization formation is crucial in reducing the risk of secondary rupture. The process of graft ligamentization and tendon bone healing after autologous ACLR has received widespread attention [10]. Ligamentosis of grafts is a long‐term process, which includes ligament formation of joint cavity segments and tendon bone healing [11]. More and more evidence suggests that the femoral side of the graft is the “weak link” in the healing of ACLR hamstring tendon autografts. One year after surgery, the highest signal of the knee joint on magnetic resonance imaging (MRI) was located at the proximal end of the graft and within the femoral tunnel [12, 13]. Basic experimental research has shown that the remnant of the ACL has the effect of promoting ligament vascularization, synovization, and proprioceptive recovery [14, 15, 16]. Previous studies have shown that different technological approaches have a significant impact on graft ligament repair and tendon bone healing. Wang et al. conducted a meta‐analysis to evaluate postoperative functional recovery and graft ligament repair in patients undergoing remnant‐preserving ACL reconstruction. The study showed no significant difference compared to simple ACLR [17]. The study conducted by Takahashi et al. showed that preserving the ACL remnant for ACL reconstruction promotes graft ligament formation [18]. However, Naraoka et al. did not find evidence that it promotes the maturation of grafts [19]. At present, whether preserving the remnant to reconstruct the ACL promotes the process of graft ligamentization is still a controversial topic. Most literature has studied the impact of preserving residual reconstruction on clinical function [20, 21, 22]. However, there is little research on the ligament formation of grafts after reconstruction surgery.

At present, the majority of studies on remnant‐preserving ACL reconstruction only use methods that simply preserve remnant. The lack of tension in the remnant may lead to absorption, and a longer remnant may also cause intercondylar fossa impact, which cannot play the role that the remnant should play.

Our research team believes that tension preservation is necessary for remnant‐preserving ACLR. Tension suspension fixation should be performed on Sherman Type I or II [23] and above remnant to preserve ACLR and cover the femoral tunnel opening. This may reduce the expansion of the femoral tunnel due to joint fluid infiltration into the bone tunnel [24], and the transplanted ligament should be wrapped around the suspended tension fixation remnant, which may promote the vascularization of the graft and facilitate the recovery of knee joint function. Many studies use MRI to evaluate graft ligament formation, which is a reliable method for assessing graft ligament formation and tendon‐bone healing [25, 26].

Our previous 2‐year retrospective follow‐up study demonstrated [27] that for Sherman Type II ACL injuries, tension‐suspension remnant‐preserving reconstruction yielded superior graft ligamentization and revascularization, as well as better International Knee Documentation Committee (IKDC) and Lysholm functional scores, compared with non‐remnant‐preserving anatomical reconstruction. Diffusion tensor imaging (DTI) further verified higher microstructural maturity of grafts in the remnant‐preserving group. Nevertheless, that study only assessed MRI signal‐noise quotient (SNQ) and DTI parameters of grafts at 6, 12, and 24 months postoperative follow‐ups. It failed to characterize graft‐healing features in the very‐early postoperative phase and did not evaluate femoral tunnel enlargement, an important postoperative complication.

Therefore, assuming that “Tension Suspension” Remnant‐Preserving ACL reconstruction promotes graft ligament formation and tendon‐bone healing, this would be a recommended new surgical approach that plays an important role in promoting early return to sport, reducing graft ligament rupture, and minimizing bone tunnel enlargement, further improving knee joint function in patients after ACLR, and reducing the significant medical burden caused by graft rupture. However, there is still a lack of comparative research on the specific effects of this surgical technique compared to traditional ACLR. The purpose of this study is to compare the differences in graft ligamentization and femoral tunnel enlargement between ACLR with “tension suspension” remnant‐preserving and single‐bundle ACL anatomical reconstruction, in order to provide further theoretical support. The specific objectives of this study were as follows: (i) to compare the early graft ligamentization process evaluated by MRI signal‐noise quotient (SNQ) between the tension‐suspension remnant‐preserving group and the non‐remnant‐preserving group, and to verify whether this novel technique can accelerate early graft maturation. (ii) To compare the degree of femoral tunnel enlargement between the two groups at different postoperative follow‐up time points and to confirm the superiority of the tension‐suspension technique in reducing postoperative bone tunnel dilatation. (iii) To assess postoperative knee functional outcomes and analyze their correlation with graft SNQ values, thereby providing evidence for postoperative rehabilitation guidance and further clinical promotion of this technique.

The present study differs substantially from our previous work. The prior study enrolled only patients with Sherman Type II remnants and conducted follow‐up up to 2 years postoperatively. By combining SNQ and diffusion tensor imaging (DTI) measurements, it confirmed that tension‐suspension remnant‐preserving reconstruction improves mid‐term graft healing and knee joint function; however, it did not evaluate very‐early‐phase graft healing or femoral tunnel enlargement. By contrast, the current study includes patients with both Sherman Type I and Type II remnants, focuses on the early postoperative period of 3–6 months, adds the indicator of femoral tunnel enlargement, and investigates the correlation between clinical function and graft SNQ values.

## 2 Materials and Method

### 2.1 Participants

Ethical approval for this study was obtained from the regional ethics committee, Approved by the Ethics Review Committee (Approval No. 2024‐346‐01YJ). We reviewed and analyzed the patient information collected by two physicians from the hospital information system HIS and arthroscopic electronic video system HD during the period from December 2021 to June 2023 for ACLR performed by the same senior chief surgeon in the sports medicine department.

The inclusion criteria for this retrospective cohort study include (1) a clear history of trauma, (2) diagnosis of ACL rupture under arthroscopy, (3) assessment of Sherman Type I or II ACL stump under arthroscopy with good synovial coverage, and (4) treatment with autologous hamstring tendon ACL stump suspension tension fixation and reconstruction or single bundle ACL anatomical reconstruction. Exclusion criteria: (1) history of knee joint surgery, (2) multiple ligament injuries or fractures near the knee joint, (3) failure to complete the subjective knee joint function score 6 months after surgery, (4) failure to undergo 3.0 T MRI at hospital 3 and 6 months after ACLR surgery, (5) severe knee joint disease or systemic disease (Kellgren Lawrence grading: III–IV osteoarthritis, rheumatoid arthritis, etc.), (6) postoperative infection. Patients meeting the above criteria were divided into two groups according to their surgical approaches: the Preserving Remnant ACLR group (PR ACLR group) and the Non Preserving Remnant ACLR group (NPR ACLR group). The patient inclusion process diagram is shown in Figure 1.

**FIGURE 1:**
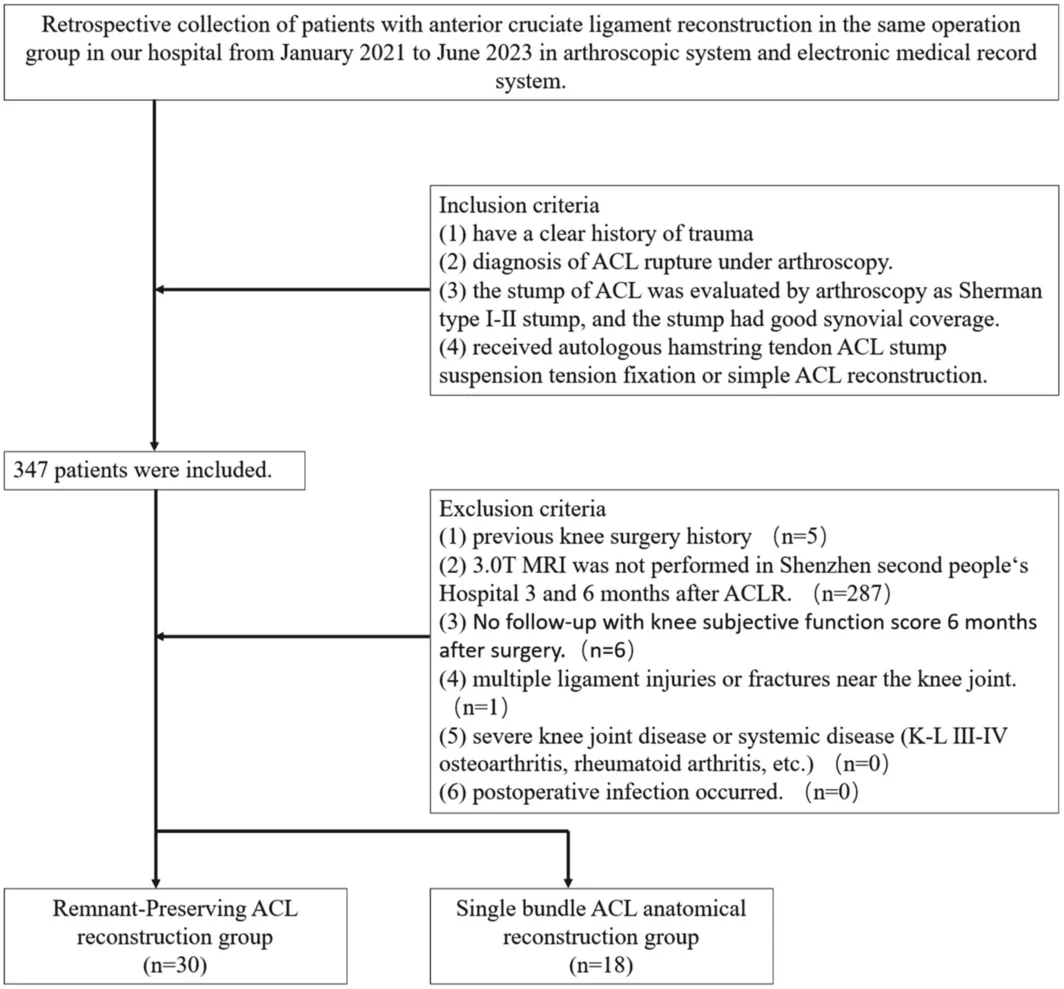
Patient inclusion process diagram.

### 2.2 Surgical Technique

Tension, suspension remnant preservation reconstruction: (1) arthroscopic assessment of the remnant was Sherman Il and above, and the femoral insertion was selected below the lateral intercondylar ridge (resident ridge) and positioned with the resident ridge as the center of the bone tunnel; to protect the tibial insertion of the ACL, the tibial insertion was selected slightly posterior to the ACL remnant, close to the center of the PL bundle (2) Using a shoulder joint suture grasper, 2 stitches of high‐strength suture were placed proximal to the ACL remnant (continuous suture method), and the high‐strength suture was pulled through the tibial tunnel with a wire penetrating grasper and threaded into the hole of the Endobutton. (3) Two strands of suture are formed through the Endobutton and folded in half as the tension‐reducing suture, which passes through the bone tunnel with the graft, the tension‐reducing suture is gradually tightened at the remnant while maintaining tension, and uses the shoulder joint knot pusher to tie and fix the knot at the surface of the Endobutton (Figure 2). Non‐remnant preservation anatomical reconstruction: the ACL remnant was excised to fully expose the femoral and tibial insertions, with the femoral insertion chosen at the anatomic center point below the resident ridge and slightly posterior to the lateral bifurcate ridge, and the tibial insertion chosen at the center of the tibial remnant, All patients were fixed with the femoral Endobutton and the tibial peek compression screw (Smith & Nephew Inc.).

**FIGURE 2:**
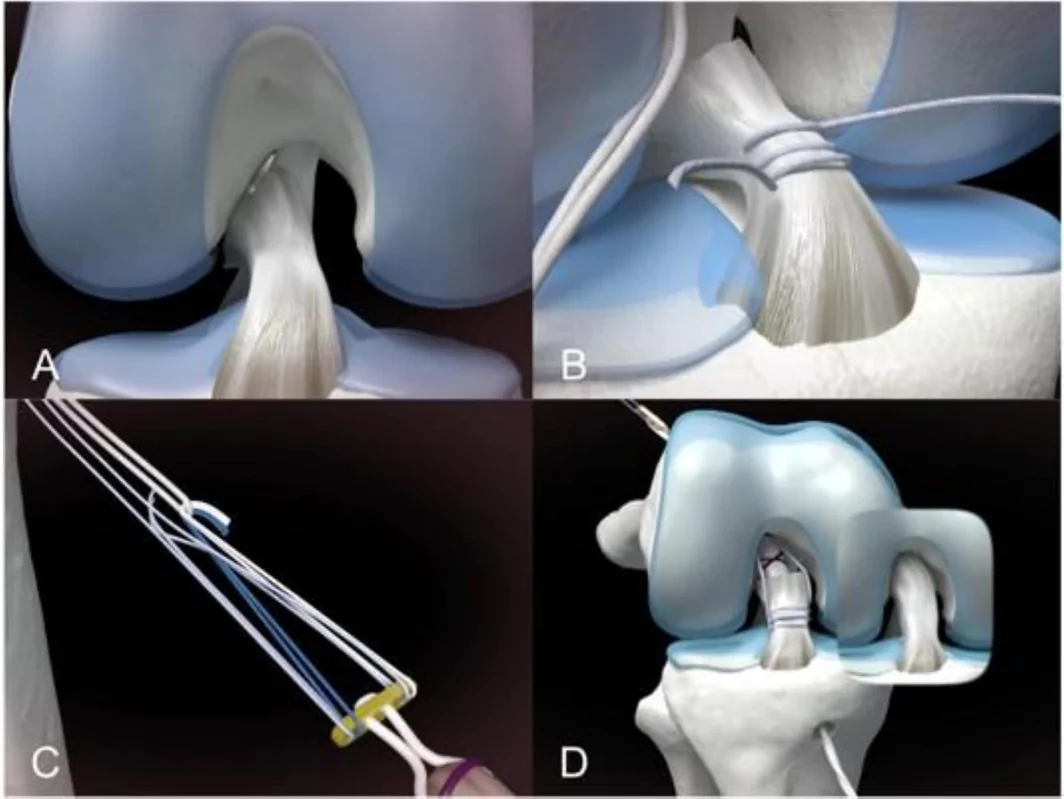
(A) Arthroscopic appearance of the anterior cruciate ligament (ACL) remnant; (B) sutureat the proximal side of the remnant where the synovium is intact. Continuous suture closure of the remnant; (C) two strands of high‐strength sutures were threaded through the Endobutton holes using a wire‐penetrating grasper via the tibial tunnel. (D) The sutures were then folded to serve as tension‐reducing sutures. These sutures, along with the graft, were passed through the bone tunnels. Under maintained tension, the tension‐reducing sutures were progressively tightened at the remnant site to ensure graft stability.

### 2.3 Rehabilitation Protocol

Postoperatively, patients performed straight leg raising and ankle pump exercises in bed, and started active knee flexion and passive knee extension training with a knee brace after 72 h. Active knee flexion and passive knee extension training continued from 4 to 12 weeks, and the knee flexion angle gradually increased to 90°–120° at 4 weeks, and quadriceps muscle strength exercises were strengthened. Full range of motion of the knee joint is possible at 3–6 months, depending on the patient's tolerance to continue weight‐bearing walking, with a gradual increase in activity and avoiding strenuous exercise; swimming and cycling are possible at 6 months after surgery, jogging resumes at 10 months, and participation in antagonistic sports is possible after 1.5 years.

### 2.4 MRI Protocol

MRI (Siemens, Germany) was performed on the affected knee at 3 months and 6 months postoperatively. Proton density‐weighted sequence (PD sequence) was adopted for scanning. The scanning parameters were set as follows: repetition time (TR) = 2700 ms, echo time (TE) = 40 ms, slice thickness = 3 mm, and field of view (FOV) = 160 mm × 160 mm. All scans were performed in the oblique sagittal plane. The scanning planes were adjusted to be parallel to the long axis of the ACL. The standardized scanning protocol was implemented by the same operator with consistent imaging conditions throughout the whole process, ensuring the clinical and experimental repeatability of imaging observation results. We focus on detecting macroscopic structures, including bone, ligaments, and meniscus, rather than microscopic structures, such as cartilage or synovium.

### 2.5 Signal/Noise Quatient (SNQ) Measurement

All included patients underwent 3.0T MRI in our hospital at 3 months and 6 months after surgery to evaluate graft maturity and femoral tunnel diameter. Intra‐articular ACL grafts were divided into three zones: proximal, middle, and distal. As described in previous studies [28], intra‐articular grafts were divided into three equally spaced zones: the Region of Interest (ROI) of the distal graft was at the proximal end of the tibial tunnel, and the distal edge was aligned with the tibial joint; the intermediate graft ROI was aligned to the anterior distal intercondylar notch; proximal graft ROI was close to the femoral tunnel opening. All ROIs were obtained with signal intensity (SI) based on an area of 10.0 ± 2.0 mm^2^, SNQ = (SI of autohamstring tendon graft—SI of quadriceps tendon)/background SI.

The SNQ of the three regions on the proton density image was used to determine graft maturity. A musculoskeletal radiologist with 5 years of experience was able to manually map the ROI of the intraarticular part of the ACL graft, including the proximal, middle, and distal regions of the graft. The background signal was measured at the lower pole of the patella and the anterior area of the patella, and the quadriceps tendon signal was measured within 5 cm of the upper pole of the patella (Figure 3).

**FIGURE 3:**
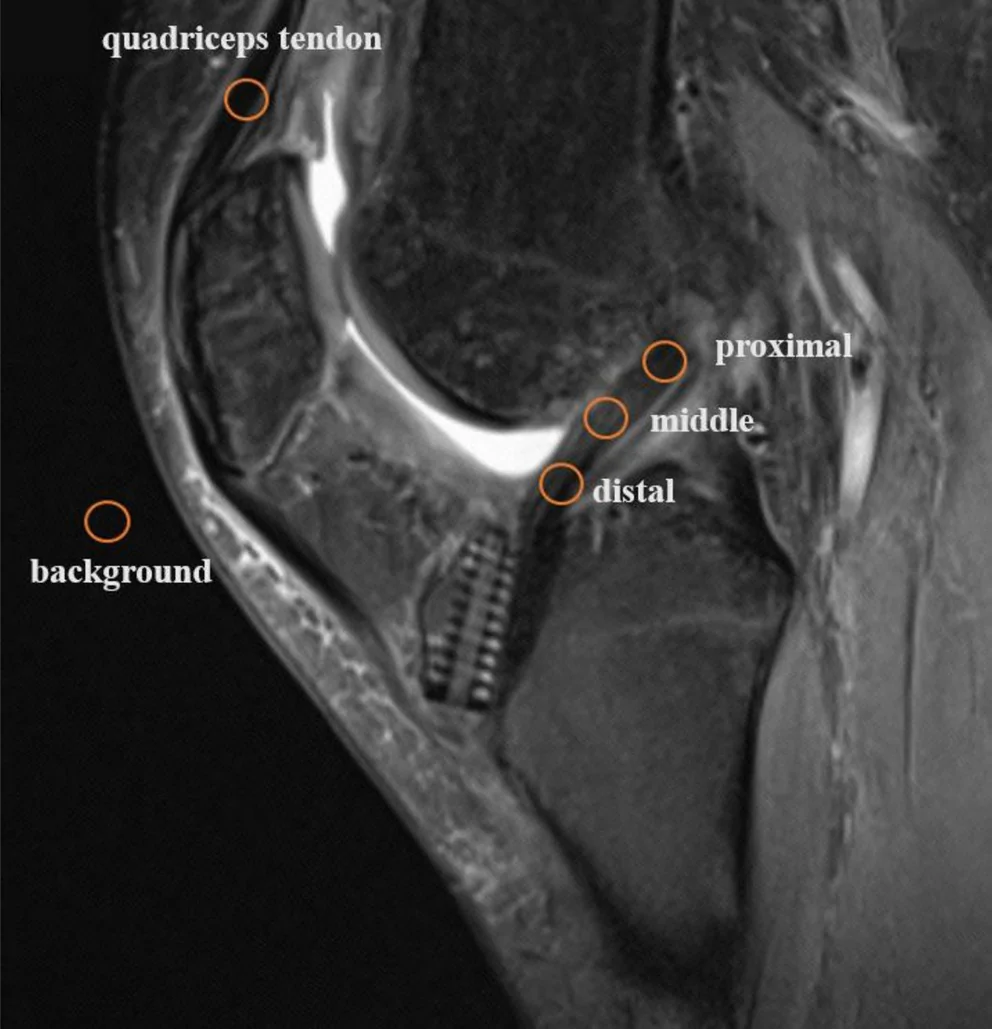
Maturity evaluation of autograft in MRI images. SI (yellow circle) of the autogenous hamstring graft was obtained at the proximal, middle1, and distal segments of the graft. SNQ = (SI of autogenous hamstring tendon graft—SI of quadriceps tendon)/background SI. SI: signal intensity, SNQ: signal noise quotient.

The diameter of the femoral tunnel was examined and measured by PACS system, the image review system of the hospital. The knee joints of all patients were examined by MRI at 3 and 6 months after surgery. The expansion of the bone tunnel was evaluated by measuring the width of the bone tunnel on the MRI scan image perpendicular to the femoral tunnel at sagittal position. The study showed that the femoral tunnel enlargement mainly occurred within 5 mm from the femoral tunnel opening [29]. Thus, we measured positions 5.0 ± 0.1 mm from the distal opening of the femoral tunnel (Figure 4). All bone tunnel measurements were performed by the same experienced orthopedic surgeon. The MRI data obtained at follow‐up were compared with the diameter of the bone tunnel drilled during the operation. The relative expansion rate is measured using the following expression: (Measurement value (*d*) − intraoperative hole diameter)/intraoperative hole diameter.

**FIGURE 4:**
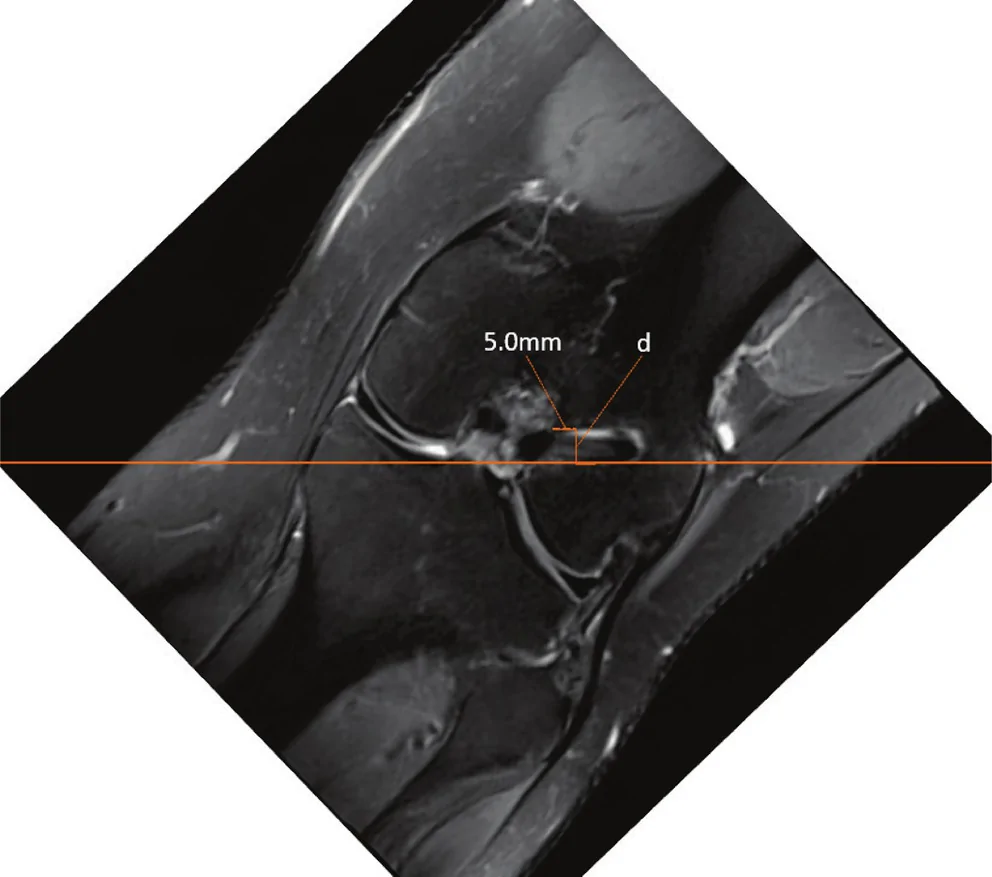
Measurement of femoral tunnel diameter *d*.

### 2.6 Clinical Assessment

Assessments included the International Knee Documentation Committee (IKDC) score, Tegner score, and Lysholm activity score. These assessments were performed and recorded 6 months after surgery. The same physician was assigned to evaluate and record the knee function at 6 months after surgery following standardized training, including telephone follow‐up and questionnaire completion.

### 2.7 Statistical Analysis

The variables of normal distribution are reported as mean ± standard deviation, the variables of non‐normal distribution are reported as median (percentage), and the descriptive data are expressed as the mean ± standard deviation of normal distribution data and the median (percentage) of non‐normal data. Classification variables of frequency and percentage.

Firstly, the normal distribution of all the measured parameters is checked by Kolmogorov and Smirnov test. Independent sample *t*‐test was used to compare the differences between the two groups. Independent sample *t*‐test was used to compare the graft signal between 3 ROI, the change value of femoral tunnel diameter and the subjective function score of knee joint 6 months after operation. The differences of non‐normal distribution parameters between the two groups were compared by Mann–Whitney *U* analysis. Mann–Whitney *U* test was used to compare the graft signal, the diameter change of femoral tunnel and the subjective function score of knee joint 6 months after operation between the three ROI. The *p* value less than 0.05 was regarded as statistically significant. Analysis of covariance (ANCOVA) was used. With the interval from injury to surgery (week) as the covariate, the mean standardized SNQ values and femoral tunnel diameter expansion rates were compared between the simple reconstruction group and remnant‐preserving reconstruction group at 3 and 6 months postoperatively. Levene's test was performed to assess homogeneity of variance before statistical analysis. All tests were two‐tailed, and a *p* value less than 0.05 was considered statistically significant. Spearman rank correlation was used to calculate the correlation between ligament graft signal and knee joint function score.

We used SPSS (version 23.0, IBM Corp., Armonk, NY) to assess test–retest reliability, with the same rater measuring the same subjects twice. A two‐way random‐effects model (absolute agreement) was applied, and we report the single‐measure intraclass correlation coefficient (ICC(2,1)). ICC values below 0.50 indicate poor reliability, 0.50–0.75 moderate, 0.75–0.90 good, and above 0.90 excellent. We also calculated 95% confidence intervals. All tests were two‐tailed with *α* = 0.05 [30].

Generate using SPSS software (IBM SPSS Statistics for Windows, version 23.0, IBM), Easestats software (www.Empowerstats.com), and R software.

## 3 Results

A total of 48 patients were enrolled in this study and were divided into two groups: NPR ACLR group (*n* = 18) and PR ACLR group (*n* = 30). As shown in Table 1, there were no significant differences in age, gender, body mass index (BMI), and graft diameter between the two groups of patients (all *p*‐values were greater than 0.05). The average age of the NPR ACLR group was 27.83 ± 8.45 years, while the PR ACLR group was 29.73 ± 7.87 years (*p* = 0.417). There was no significant difference in gender distribution between the two groups, with males accounting for 72.22% and females accounting for 27.78% in the NPR ACLR group; the PR ACLR group had 83.33% males and 16.67% females (*p* = 0.296). In addition, the average BMI of the NPR ACLR group was 24.93 ± 3.97, while that of the PR ACLR group was 23.90 ± 2.82 (*p* = 0.463). The graft diameter was 7.83 ± 0.57 mm in the NPR ACLR group and 8.08 ± 0.47 mm in the PR ACLR group (*p* = 0.108), with a median graft diameter of 8 mm. Nine (50.0%) patients in the NPR ACLR group and 18 (60.0%) patients in the PR ACLR group received tendon transplants with a graft diameter of 8.0 mm.

**TABLE 1:** Patient characteristics.

	NPR ACLR (*n* = 18)	PR ACLR (*n* = 30)	*p*‐value*
Age (years)	27.83 ± 8.45	29.73 ± 7.87	0.417
Gender			
Man	13 (72.22%)	25 (83.33%)	
Female	5 (27.78%)	5 (16.67%)	
BMI (kg/m^2^)	24.93 ± 3.97	23.90 ± 2.82	0.463
Graft diameter (mm)	7.83 ± 0.57	8.08 ± 0.47	0.108
Time to injury surgery (weeks)	33.83 ± 111.80	2.53 ± 2.98	0.007
Side			0.654
Left	9 (50.00%)	13 (43.33%)	
Right	9 (50.00%)	17 (56.67%)	
Meniscus injury			0.762
No	7 (38.89%)	13 (43.33%)	
Yes	11 (61.11%)	17 (56.67%)	
Medial meniscus injury			0.582
No	15 (83.33%)	23 (76.67%)	
Yes	3 (16.67%)	7 (23.33%)	
Lateral meniscus injury			0.709
No	10 (55.56%)	15 (50.00%)	
Yes	8 (44.44%)	15 (50.00%)	
Injured while participating in sports			0.709
No	1 (5.56%)	1 (3.33%)	
Yes	17 (94.44%)	29 (96.67%)	
Type of sport during injury			0.706
Basketball	7 (38.89%)	15 (50.00%)	
Football	2 (11.11%)	3 (10.00%)	
Badminton	0 (0.00%)	1 (3.33%)	
Sprain/Trauma	9 (50.00%)	11 (36.67%)	

*Note:* Results in the table: mean SD/*N* (%).

There is a significant difference in the preoperative injury characteristics between the NPR ACLR group and the PR ACLR group. The average time from injury to surgery in the NPR ACLR group was 33.83 ± 111.80 weeks, while in the PR ACLR group it was 2.53 ± 2.98 weeks (*p* = 0.007), and this difference was statistically significant. There was no significant difference in the distribution of left and right ACL injuries between the two groups (*p* = 0.654). For meniscus injuries, 11 individuals (61.11%) in the NPR ACLR group had meniscus injuries, while 17 individuals (56.67%) in the PR ACLR group had meniscus injuries, and no significant difference was found between the two groups (*p* = 0.762). In addition, there was no significant difference in the distribution of medial and lateral meniscus injuries between the NPR ACLR group and the PR ACLR group, with medial meniscus injuries (*p* = 0.582) and lateral meniscus injuries (*p* = 0.709), respectively.

Regarding the cause of injury, almost all patients had sports injuries, and there was no statistically significant difference between the NPR ACLR group of 17 people (94.44%) and the PR ACLR group of 29 people (96.67%) (*p* = 0.709). In terms of injury types, there was no significant difference in the distribution of exercise types between the NPR ACLR group and the PR ACLR group, with basketball being the most common exercise type, accounting for 38.89% and 50.00%, respectively (*p* = 0.706).

### 3.1 Reliability of Measurements

The single‐measure ICC(2,1) values were 0.829 (95% CI: 0.451–0.955) for femoral tunnel diameter, indicating good reliability, and 0.943 (95% CI: 0.801–0.985) for the SNQ value, reflecting excellent agreement.

### 3.2 Inter Group SNQ Comparison

The analysis results of the mean SNQ of the two groups of transplanted ligaments at 3 months and 6 months after surgery show that there is a significant difference in the mean SNQ of the transplanted ligaments between the two groups at 3 months after surgery (*p* < 0.05), as shown in Table 2. The 95% confidence interval of the mean graft SNQ in the PR group was evidently lower than that in the NPR group at 3 months postoperatively, indicating a statistically significant intergroup difference. This conclusion can be drawn from the significance results of Mann Whitney *U* test, Wilcoxon signed rank test, *Z*‐value, and *p*‐value. On the contrary, there was no significant difference in the mean SNQ of transplanted ligaments at 6 months after surgery (*p > 0.05*).

**TABLE 2:** SNQ values for MRI evaluation of knee joint grafts.

	PR ACLR (*n* = 30)	NPR ACLR (*n* = 18)	*p* value
3 months			
Proximal	17.5609 ± 22.8522	56.1963 ± 62.5152	0.006
Middle	14.2146 ± 14.398	56.4618 ± 58.3392	0.011
Distal mean	7.6047 ± 15.3406	35.4829 ± 47.7175	0.001
6 months			
Proximal	31.1128 ± 26.0049	38.8291 ± 40.7138	0.958
Middle	24.9489 ± 23.9969	46.4842 ± 62.153	0.302
Distal mean	17.4798 ± 21.2103	40.6427 ± 55.2176	0.058
Mean of 3 months postoperative SNQ		49.3803 ± 52.8976	0.005
Mean of 6 months postoperative SNQ	24.5138 ± 20.9714	41.9853 ± 49.2759	0.437

*Note:* Expressed as mean ± standard deviation.

At 3 months after surgery, the proximal SNQ values, middle SNQ values, distal SNQ values, and mean SNQ values of the two groups of grafts reached statistical significance, especially the distal SNQ values of the grafts at 3 months, which showed the most significant difference (*p* < 0.001). In contrast, there was no significant difference (*p* > 0.05) in the proximal and middle SNQ values of the grafts between the two groups 6 months after surgery. The difference in distal SNQ values of the graft at 6 months after surgery was close to a significant level (*p* = 0.058), indicating that there was a certain difference in the site between the two groups.

### 3.3 Comparison of Changes in Femoral Tunnel Diameter Expansion Rates Between Two Groups

As shown in Table 3, there was a significant statistical difference (*p* < 0.05) in the changes in femoral tunnel diameter expansion rates between the two groups on MRI at 3 months after surgery, while there was no significant difference (*p* > 0.05) in the 6‐month group. At 3 months after surgery, the relative enlargement rate of the femoral tunnel in the PR group was markedly reduced compared with the NPR group.

**TABLE 3:** Comparison of changes in femoral tunnel diameter expansion rates between the two groups.

	PR ACLR (*n* = 30)	NPR ACLR (*n* = 18)	*p*
3 months after surgery	20.28 ± 16.02	39.51 ± 10.75	0.024
6 months after surgery	31.13 ± 25.59	41.94 ± 15.57	0.124

### 3.4 Comparison of Primary Outcomes After Adjustment for Baseline Time Differences

MANCOVA revealed a clear overall effect of surgical technique (Wilks' Λ = 0.119, *p* < 0.001). At 3 months, the NPR ACLR showed higher adjusted means than the PR ACLR for both femoral tunnel diameter expansion rates (0.402 vs. 0.199, *F*(1,45) = 96.50, *p* < 0.001) and SNQ (51.53 vs. 12.16, *F*(1,45) = 22.36, *p* < 0.001). At 6 months, there were no significant differences between the two groups (*p* = 0.139 and *p* = 0.090, respectively). The covariate (time from injury to surgery) was not significant (*p* = 0.408).

### 3.5 Comparison of Subjective Functional Scores Reported by Patients 6 Months After Surgery

As shown in Table 4, this study found that there was no significant statistical difference (*p* > 0.05) between the PR ACLR group and the NPR ACLR group in terms of Lysholm score, IKDC score, and Tegner score.

**TABLE 4:** Comparison of subjective functional scores reported by patients 6 months after surgery.

	PR ACLR (*n* = 30)	NPR ACLR (*n* = 18)	*p*
Lysholm	87.33 ± 7.443	90.06 ± 5.330	0.148
IKDC	81.40 ± 7.389	82.44 ± 4.162	0.534
Tegner	4.27 ± 0.828	4.28 ± 0.826	0.964

### 3.6 The Correlation Between Transplanted Ligament Signals and Knee Joint Function Scores

Spearman correlation was used to analyze the relationship among Lysholm, IKDC, Tegner, and the mean value of SNQ at 3 and 6 months after operation. The results showed that the correlation coefficient between the Tegner rating and the mean value of SNQ at 3 months after operation was 0.302, and the significance was 0.037 (*p* < 0.05), indicating that there was a moderate positive correlation between them. The correlation coefficient between the Tegner rating and the mean SNQ at 6 months after surgery is 0.356, with a significance of 0.013 (*p* < 0.05), indicating a significant positive correlation between the two.

## 4 Discussion

We found that patients receiving tension suspension remnant‐preserving ACL reconstruction achieved substantially better early outcomes at 3 months postoperatively. Specifically, this cohort showed significantly lower graft SNQ values, particularly in the distal graft segment, alongside a markedly reduced rate of femoral tunnel enlargement, confirming superior early graft maturation and effective inhibition of tunnel dilatation. By the 6‐month follow‐up, intergroup differences in both graft ligamentization and femoral tunnel widening had diminished. No statistically significant differences were detected in postoperative Lysholm, IKDC, or Tegner functional scores between the two groups at this time point. Correlation analysis identified a moderate but significant positive correlation between graft SNQ values and postoperative Tegner sport scores, confirming the close association between early graft healing quality and subjective knee sports performance.

### 4.1 Effects of Tension Suspension Remnant‐Preserving on Early Graft Ligamentization

The most important finding of this study was that during the MRI scan 3 months after operation, there was a significant difference in the overall mean value of SNQ of intra‐articular grafts between the two groups. ACLR with tension‐preserving stump suspension fixation was faster than that of anatomical single bundle ACLR, and the enlargement of the femoral tunnel was significantly smaller. Consistent with our hypothesis, in the early stage of graft ligament formation, the tension maintaining residual suspension fixation ACLR group had better early ligament formation than the anatomical single bundle ACLR group. However, the difference at 3 months postoperatively did not translate into differences in intra‐articular ligament formation and reduction of femoral tunnel enlargement at 6 months postoperatively.

There was a significant difference in the mean value of graft SNQ 3 months after operation between the two groups. In the study of preserving Remnant‐Preserving ACLR to promote graft ligamentization. Okutan et al. found in their study exploring the influencing factors of ACL graft ligament formation that whether to preserve the ACL stump is the most important factor affecting graft healing after ACLR [31]. Our study, similar to previous researchers, preserves the reconstruction of residual ACL to promote graft maturation [18, 32]. However, some studies have shown that there is no significant difference between ACLR with and without remnant preservation [17, 33]. At present, it is controversial whether the preservation of remnant reconstruction can promote the process of graft ligamentization. This study shows that in MRI scan 3 months after operation, ACLR with tension‐preserving remnant suspension is better than single‐beam ACLR. We think it is related to the synovial coverage of the remnant and the treatment of the remnant. When choosing whether to preserve ACL remnant reconstruction, we chose a remnant with good synovial coverage under arthroscopy, Kim et al. showed that preserving the remnant during ACLR but poor synovial coverage was not beneficial. Preserving the remnant with good synovial coverage had a positive effect on graft synovialization and the maintenance of graft integrity [34], but this effect was not observed in patients with poor synovial coverage [34]. The extent of synovial coverage should be taken into account when deciding whether to retain the residue. Experimental studies have shown that there are mesenchymal stem cells with multiple differentiation ability in the remnant of ACL [34]. CD34 positive cells derived from ACL residues contribute to tendon bone healing after ACLR [15], Therefore, the ACL remnant with good synovial coverage can be reconstructed by preserving the remnant to give play to the greatest advantage of the remnant.

At present, the methods of remnant retention are mainly divided into three types [17, 35], tensile disability protection [36], non‐tension disability protection, and cuff‐like disability protection [37, 38]. We adopted the method of preserving the remnant to protect the remnant by tension, and we included the remnant of Sherman Type II and above to be suspended and fixed with tension (Figure 2). On the one hand, it increased the mechanical stability of the graft, but unfortunately, our retrospective cohort did not study the indicators related to knee joint stability after tension fixation and disability preservation of ACLR. In the future, we can further study the effect of disability‐preserving reconstruction with suspension tension fixation on postoperative knee joint stability in Sherman Type I or II remnant. On the other hand, the remnant of Sherman Type II and above can be covered on the graft by suspension tension fixation, so that the remnant derived factors that promote the vascularization, tendon band, and tendon healing of the graft can play the best role [15].

In the MRI evaluation 3 months after operation, the most significant difference in intra‐articular graft between the two groups was in the proximal end of the graft (*p* = 0.006) and the distal end of the graft (*p* = 0.001), which indicated that the PR ACLR group had better ligamentization in the proximal and distal end of the graft. There are several explanations for this better graft remodeling in terms of the difference in the proximal end of the graft. one is that we think it is related to the coverage of the remnant, because we choose the Sherman I or II remnant, which can often cover the proximal end of the graft and give full play to the role of the remnant during suspension tension fixation. On the one hand, the difference of the distal end of the graft is related to the coverage of the remnant, and the other is that the blood supply of the remnant of ACL is better near the tibial end, so that the coverage near the remnant provides better vascularization for the distal end of the graft. This may be similar to the reason observed by Nicolas et al. [39] in ACLR with preservation of distal attachment of the semitendinosus muscle, which is related to the improvement of graft ligament formation, as there is blood supply at the semitendinosus insertion point, providing better vascularization for the graft.

However, the advantage of graft toughening at 3 months after operation could not be observed at 6 months after operation. Research has shown that the healing factors of residual tissue after ACL injury are negatively correlated with the time after injury [14, 40]. The effectiveness of ACL residual healing factors decreases over time [41]. Nayak et al. observed the ultrastructure and histological changes of the residual tissue through transmission electron microscopy and immunohistochemistry. The study showed that the density of blood vessels and fibroblasts was highest between 12 and 20 weeks after injury [40]. It has also been demonstrated in animal experiments that early transplantation of ACL residual tissue after injury can accelerate and promote tendon bone healing. Clinical relevance is reflected in the impact of injury to surgical interval on the healing potential of human ACL derived cells [42]. At 6 months postoperatively, there was no significant difference in the advantage of graft ligament formation between the PR ACLR group and the NPR ACLR group (PR ACLR: 24.51 ± 20.97 VS NPR ACLR: 41.98 ± 49.27, *p* = 0.437). The most likely reason is that the healing factor of the ACL remnant after injury gradually reduces its effectiveness in promoting vascularization, synovization, and ligament formation over time [43]. The advantages provided by the reconstruction of the retained remnant with tension fixation after 3 months of surgery did not translate into advantages at 6 months. There may be several reasons for this. Firstly, due to the lack of blood supply, the remnant is gradually absorbed over time. Secondly, as the rehabilitation process progresses, the patient's activity level increases, which may lead to the failure of suspension fixation. The third method is to use suturing to suspend and fix Sherman Type I or II remnant, forming mechanical fixation. The remnant with tension fixation serves as reinforcement structures for ACLR, without biological healing. As the activity increases, repeated cutting of the suture may lead to the failure of high‐strength thread suspension and accelerate the absorption of the remnant.

Notably, in the PR group of this study, the MRI SNQ values increased from 13.12 at 3 months postoperatively to 24.51 at 6 months postoperatively, indicating that signal intensity rose over time. This finding is inconsistent with the traditional view that graft maturation is accompanied by decreased signal intensity. According to the pathophysiological process of ligament graft healing, the period from 3 to 6 months after surgery remains an active stage of graft revascularization, cell proliferation and collagen remodeling. Vigorous neovascularization and cell proliferation during this phase can lead to edema‐like high signals on MRI, resulting in a transient elevation of SNQ values, which does not indicate graft immaturity or abnormal inflammatory response [11]. Accordingly, such increased signals are more likely to reflect the active biological status of early graft revascularization and tissue remodeling, rather than simple reactive edema or pathological lesions. Nevertheless, due to the absence of histological evidence, it is currently impossible to fully distinguish whether these signal changes are physiological alterations related to remodeling or local edema. Further studies with longer follow‐up periods and multimodal imaging examinations are needed to clarify its clinical significance in the future.

Another point worth noting is that there was a marked baseline imbalance in injury‐to‐surgery intervals between the PR and NPR groups (2.53 ± 2.98 weeks vs. 33.83 ± 111.80 weeks, *p* = 0.007). Preclinical data show [14, 40, 43] that ACL remnant pro‐healing factors, vascular density and mesenchymal stem‐cell activity decline over time after injury; acute remnants exert stronger effects promoting graft revascularization and synovialization, while chronic stumps tend to undergo fibrous degeneration and reduced cellular activity. Thus, the better early graft ligamentization (lower SNQ) in the PR group at 3 months could be partially confounded by shorter preoperative injury duration. Although multivariate ANCOVA adjusting for injury‐to‐surgery time retained significant inter‐group differences for SNQ and femoral tunnel expansion, with the covariate showing no independent effect (*p* = 0.408), residual biological confounding cannot be fully excluded. Acute remnants may intrinsically enhance graft remodeling and synergize with the tension‐suspension procedure. Consequently, the observed early healing advantages of this technique should be interpreted prudently, as some of the beneficial effect may derive from the native biological characteristics of acute‐phase remnants rather than the surgical approach alone.

### 4.2 Inhibitory Effect of Tension Suspension Remnant‐Preserving on Early Femoral Tunnel Enlargement

Bone tunnel enlargement is a common complication after ACLR surgery [44]. The reason for the enlargement of bone tunnel after operation is not clear. The enlargement of bone tunnel is related to the decision of revision operation [45]. The enlargement of bone tunnel is a risk factor for the failure of ACL graft, and the enlargement of bone tunnel is related to the stability of knee joint [44]. Related studies have shown that the expansion of bone tunnels is the result of a combination of mechanical and biological factors [46]. Biological factors such as degradation of absorbable implants, diffusion of synovial fluid into tunnels, and poor bone quality are considered potential biological factors [47]. Other biological factors include cytokines (interleukin‐1, interleukin‐6, tumor necrosis factor, nitrogen monoxide, etc.) [48], inflammatory mediators, graft type, bone quality, and bone necrosis caused by drilling bone tunnels [47, 49]. Mechanical factors, including the fixation method of the graft and the movement of the graft within the tunnel, are considered to play an important role [50], especially when performing ACL single bundle reconstruction, the use of femoral loops for suspension fixation can lead to mechanical factors such as “bungee effect” or “windshield wiper effect” causing micro movement of the graft in the bone tunnel [50, 51]. Other reasons for femoral tunnel enlargement include bone tunnel localization [52], graft fixation methods [53], early postoperative active rehabilitation [51], and coronal graft curvature angle. The research of Chiang et al. showed that the enlargement of bone tunnel mainly occurred within 6 months after ACLR [54], and some scholars thought that the enlargement of bone tunnel occurred within 3 months after operation [55].

Our study showed that there was a significant difference in the enlargement of femoral tunnel 3 months after operation, and the enlargement of femoral tunnel in PR ACLR group was smaller than that in NPR ACLR group (PR: 1.5567 ± 1.38407 vs. NPR: 3.0889 ± 0.85260, *p* = 0.000). Koyama et al. observed the effect of a new type of remnant‐preserving anatomical double‐beam reconstruction on the enlargement of femoral and tibial tunnels after ACLR. Their studies have shown that this new type of disability‐preserving reconstruction can effectively reduce the enlargement of femoral and tibial tunnels 1 year after operation [56]. Li et al. reduce the enlargement of the femoral tunnel after ACLR by preserving the remnant on the femoral side [57]. The meta‐analysis of Tie et al. concluded that preserving the remnant of ACL to reconstruct the ACL can reduce the expansion of tibial tunnel [58]. The remnant we retained is Sherman II and above, and the remnant is fixed by suspension tension to maintain the tension of the remnant, which can partially cover the femoral tunnel orifice, one is to reduce the infiltration of articular fluid into the bone tunnel orifice, and the other is the remnant healing factor to promote tendon‐bone healing [15]. Third, the tensioned remnant can enhance the ACL graft, reduce the mechanical load of the graft, and then reduce the micromovement of the graft in the bone tunnel, and the degree of “bungee effect” or “windshield wiper effect” of the graft is reduced. Liu et al. measured the diameter of the ACLR tunnel using a three‐dimensional model of computed tomography data. Their study showed that the femoral and tibial tunnels exhibited eccentric widening, which gradually stabilized 1 year after ACLR [44]. Devitt et al.'s imaging follow‐up study on bone tunnel enlargement after suspending and fixing autologous hamstring tendon ACLR showed that the width of the bone tunnel did not increase after 4 months [59]. Therefore, the enlargement of the bone tunnel is more significant in the early postoperative period. Our study showed that although there was no significant difference in reducing femoral tunnel enlargement between the PR ACLR group and the anatomical single bundle reconstruction group at 6 months after surgery(PR ACLR: 2.4900 ± 2.18543 vs. NPR ACLR:3.2611 ± 1.18428, *p* = 0.120). However, the PR group still has a certain advantage in reducing femoral tunnel enlargement, unfortunately, this advantage does not have a statistically significant difference. There may be the following reasons: firstly, the residual healing factor is negatively correlated with time; Secondly, during the period of 3–6 months after surgery, the expansion of the bone tunnel is not the most significant period, and the significant expansion of the bone tunnel occurs before 4 months after surgery [59].

### 4.3 Correlation Between Graft Healing Status and Postoperative Knee Functional Recovery

This study showed that there was no significant difference in the score of knee joint subjective function between the two groups in the early stage after operation. This is consistent with the meta‐analysis findings of Wang et al. [17] and Tie et al. [58], which showed that residual preservation of ACLR resulted in similar postoperative clinical scores compared to single bundle anatomical ACLR. Annear et al. conducted a randomized controlled trial to compare the 10‐year outcomes of the PR group and the NPR group. Their study showed no significant differences in knee joint function and recurrence rate between the two groups [60]. Choi et al. compared the clinical outcomes of single bundle ACLR using the residual reconstruction technique with the traditional ACLR technique, and did not observe significant differences in IKDC and Lysholm scores between the two techniques [61]. Franciozi et al. [62] compared residual preservation reconstruction with nonresidual preservation reconstruction, and found that residual preservation was not associated with functional improvement, which is consistent with our research findings. Huang et al.'s [63] study showed that patients treated with remnant preservation reconstruction techniques may achieve better outcomes in terms of graft injury and recovery to pre injury exercise levels, but there is no difference in overall recovery exercise. In a study comparing the retention of residual length and clinical efficacy by Liu et al. [64], it was found that patients with ACLR who retained more than one‐third of the original residual length had higher activity levels 12 months after surgery, consistent with the study by Kim et al. Their research showed that compared with traditional ACLR, retaining more than two‐thirds of the residual length was more effective in improving activity related clinical outcomes than not retaining residual reconstruction [65]. According to the literature reviewed at present, in the study of retained remnant reconstruction ACL and non‐reserved reconstruction ACL, whether there is a relationship between disability preservation reconstruction ACL and knee joint functional outcome is controversial. However, whether there is a correlation between residual reconstruction and knee joint function outcomes may be related to the way the remnant is preserved. Previous studies have emphasized the importance of preserving remnant tension in remnant reconstruction [36, 66]. In this study, we adopted the method of retaining and preserving the remnant by suspending tension fixation. At the 6‐month follow‐up after surgery, no difference in subjective functional scores was observed between the two groups. This may be due to the fact that we included a population with lower levels of physical activity, non high‐level athletes, and low demand for returning to exercise; secondly, the follow‐up time is short, and the exercise of the study population is limited by rehabilitation plans. In the future, medium to long‐term follow‐up can be conducted. Third, the enrolled sample size was relatively limited, with a total of only 48 patients, including 18 cases in the NPR group. Insufficient sample size may exert certain impacts on statistical power. A small sample size is usually associated with a higher risk of Type II error, meaning genuine clinical differences between groups may fail to be detected due to inadequate statistical power. To further clarify the actual effect of ligament remnant preservation on clinical functional scores, future studies should expand the sample size and conduct multicenter trials to improve statistical power and verify intergroup differences more reliably.

In this study, Spearman correlation analysis was conducted to investigate the correlation between transplanted ligament signals and knee joint function scores. The results showed a positive correlation between Tegner score and mean SNQ, with higher postoperative exercise levels leading to a larger mean SNQ of the transplant at 6 months. Yau et al. evaluated graft ligamentization after ACLR using SNQ in a group of 565 ACLRs, and observed complete graft ligalization at 4 years postoperatively [67]. The research results of Zhang et al. showed that graft ligament ligamentization was not completed until 24 months after ACLR surgery [26]. Marcus et al. compared the SNQ of autologous hamstring tendon grafts with that of healthy individuals' ACL at 6 months postoperatively, and the results showed that the transplanted ligaments were immature at 6 months postoperatively [68]. The mean value of SNQ was positively correlated with the Tegner score, which may be due to the fact that the knee function score of our follow‐up was obtained at 6 months after operation, the graft was immature at 6 months after operation, and the biomechanical strength was insufficient. The higher the level of exercise at 6 months after operation, the greater the force on the graft, which is a potential injury factor for the graft, which may cause graft injury or reactive edema. In MRI, the mean value of SNQ increased. Therefore, especially within 6 months after ACLR, doctors should be aware that the graft is not fully matured, and the patient's return to adversarial, high‐intensity exercise increases the risk of graft re rupture. However, for competitive athletes who are clinically stable and wish to resume exercise after 6 months, performing MRI to confirm the stage of graft healing may be an option.

### 4.4 Complications of Tension Suspension Remnant‐Preserving ACLR

Although the technique of preserving residual reconstruction has obvious advantages, the complications it brings are also worthy of clinical attention. Kulczycka et al. observed through imaging studies that the main complications of this procedure are poor positioning of the femoral or tibial bone tract and collision between the graft and the femoral intercondylar fossa [69]. In addition, preserving the remnant to reconstruct ACL increases the diameter of the graft and thus increases the volume of the graft, which can easily lead to “Cyclops Syndrome,” impact between the graft and the intercondylar fossa, and limitation of knee joint extension. However, Delaloye et al. [70] and Demira ğ et al. [71] showed that retaining most of the ACL remnant was not a risk factor for “Cyclops Syndrome,” and there was no significant difference in the incidence rate of “Cyclops Syndrome” at the remnant in single bundle ACLR [72]. Suh et al. [36] suggested maintaining tension in the remnant to avoid the formation of “Cyclops Syndrome.” Sonnery et al. [73] emphasized the importance of correct femoral canal localization in effectively reducing the occurrence of complications. Kosy et al. [74] found that single bundle hamstring tendon preservation for residual reconstruction does not affect the accuracy of tibial canal localization. Liu et al. showed that there was no difference in the accuracy of ACL femoral tunnel establishment between disability preservation reconstruction technique and non‐retained remnant technique [75]. Meanwhile, Liu et al.'s research indicates that the technique of preserving the remnant does not sacrifice the ideal position of the femoral tunnel and can potentially benefit from preserving the ACL remnant [75]. In the study of accurate localization of ACL bone tunnel reconstruction with retained remnant, Zhang et al. proposed that the “apex of the deep cartilage (ADC)” localization method is a stable marker for femoral tunnel localization during ACLR with retained remnant [76]. Lee et al. proposed that ADC may be a marker for preserving ACLR through the “posterior transseptal” approach, which is located at the proximal corner of the ACL remnant and provides accurate localization advantages for the femoral tunnel during the preservation of ACLR [77]. Therefore, it is crucial to master the correct ACLR bone tunnel localization method in ACLR surgery with remnant preservation.

### 4.5 Study Strengths

The present study has several prominent strengths. First, this is the first clinical study to specifically evaluate the efficacy of the novel tension‐suspension remnant‐preserving ACL reconstruction technique, focusing on early graft ligamentization and femoral tunnel enlargement. Different from conventional tension‐free remnant‐preserving, this study confirmed the unique early healing advantages of tension‐maintained remnant wrapping, providing novel clinical evidence for optimizing remnant‐preserving strategies. Second, all postoperative MRI examinations and quantitative SNQ measurements were performed with unified scanning parameters and strict standardized ROI delineation, and excellent inter‐rater reliability was verified, ensuring high accuracy and reproducibility of the imaging quantitative results. Third, this study not only compared traditional morphological indicators (bone tunnel enlargement) and functional scores, but also innovatively analyzed the correlation between graft imaging maturity and postoperative sports function, which established a clinical reference for evaluating early graft healing status and guiding individualized postoperative rehabilitation. Fourth, strict inclusion and exclusion criteria were adopted to enroll patients with intact synovial remnant coverage, which eliminated the confounding effects of poor remnant quality and made the study conclusions more targeted and credible for clinical application.

### 4.6 Limitation

This study has several limitations. Firstly, this is a retrospective cohort study without random grouping, which is prone to selection bias and makes it difficult to balance baseline confounding factors. A major baseline imbalance existed in the injury‐to‐surgery interval between groups: patients receiving tension‐suspension remnant‐preserving reconstruction underwent surgery predominantly in the acute phase after injury, while the non‐remnant‐preserving group contained more chronic ACL rupture cases. Although analysis of covariance was used to statistically adjust for this variable, residual confounding from the differing biological quality of acute versus chronic ACL remnants cannot be completely eliminated, which may influence graft SNQ values and tunnel healing outcomes. In addition, the small enrolled sample size leads to insufficient statistical power, limiting the generalizability of the conclusions. The follow‐up period in this study was only 6 months, during which the graft remained in the weak remodeling stage with unstable biomechanical strength and collagen arrangement. The imaging findings can only reflect the early healing status but cannot determine long‐term healing quality and prognosis. Long‐term dynamic follow‐up of 12–24 months is still required. Meanwhile, the reliability and repeatability of the data were not verified, and the stability of the results needs further confirmation.

Secondly, MRI signal‐to‐noise ratio was used to evaluate graft healing and bone tunnel changes, while 3D‐CT, the gold standard for measuring bone tunnel widening, was not adopted. MRI results are susceptible to interference from surrounding tissue edema, synovial fluid, and other factors, resulting in signal deviation and limited evaluation accuracy. It can only reflect changing trends and is difficult to achieve accurate quantification.

Thirdly, the intraoperative retention of ligament remnant relied on surgeons' subjective judgment without unified objective evaluation criteria. No secondary arthroscopic exploration was performed, so the actual healing status of the graft could not be directly assessed.

Fourthly, objective outcome indicators such as KT‐1000 laxity measurement and muscle strength test were absent, leading to an incomplete evaluation system.

Fifthly, clinical functional scores were collected via telephone interviews and questionnaires, which were vulnerable to response bias caused by individual patient factors. Although educational level was balanced and follow‐up communication was standardized in both groups, subjective errors could not be completely eliminated.

Finally, this study failed to analyze the effects of comorbidities such as meniscal and cartilage injuries on postoperative efficacy, with inadequate control of confounding factors.

In future research, large‐sample prospective randomized controlled trials should be conducted with extended follow‐up duration and improved data reliability verification. Combined multiple imaging examinations and objective functional indicators, unified surgical evaluation criteria, and stratified analysis of combined injuries are recommended to further enhance the scientificity and clinical reference value of relevant studies.

## 5 Conclusion

Compared with single bundle ACL anatomical reconstruction, “Tension Suspension” Remnant‐Preserving ACL reconstruction that maintains tension has significant advantages in early graft ligamentization and femoral tunnel enlargement. In addition, subjective knee joint function may be related to transplant signal intensity.

## Author Contributions

**Jiamin Liang:** conceptualization, data curation, writing – original draft, methodology, formal analysis. **Gengen Huang:** conceptualization, writing – review and editing, investigation, methodology. **Wenhan Yang:** methodology, writing – review and editing. **Junming Wei:** methodology, data curation, writing – review and editing. **Yingzhen Yang:** methodology, visualization, writing – review and editing. **Zirong Huang:** data curation, writing – review and editing. **Weimin Zhu:** conceptualization, funding acquisition, methodology, writing – review and editing.

## Funding

This study was supported by the Guangdong Basic and Applied Basic Research Foundation (Grant No. 2023A1515010371); the Shenzhen Science and Technology Program (Grant Nos. JCYJ20210324102607021, GJHZ20220913143201002); the Clinical Research Fund of Shenzhen Second People's Hospital under Shenzhen High‐level Hospital Construction Project (Grant Nos. 2023yjlcyj030, 20243357002); the Medical‐Engineering Interdisciplinary Research Foundation of Shenzhen University (Grant No. 2023YG007); and the Shenzhen University Medical‐Educational Collaborative High‐Quality Innovation and Development Project (Grant No. YJXT20250103).

## Ethics Statement

This study was approved by the Regional Ethics Review Committee (Approval No.: 2024‐346‐01YJ). All participants provided written informed consent, and all procedures were performed in accordance with the ethical standards of the institutional research committee and the 1964 Helsinki Declaration and its later amendments.

## Conflicts of Interest

The authors declare no conflicts of interest.

## Data Availability

All raw data, tables, and figures generated and analyzed during this study are available from the corresponding author upon reasonable request. No publicly available datasets were used in this study.
